# Mapping of Chronic Pulmonary Aspergillosis in Africa

**DOI:** 10.3390/jof7100790

**Published:** 2021-09-23

**Authors:** Ronald Olum, Iriagbonse Iyabo Osaigbovo, Joseph Baruch Baluku, Jannik Stemler, Richard Kwizera, Felix Bongomin

**Affiliations:** 1 School of Medicine, College of Health Sciences, Makerere University, Kampala P.O. Box 7072, Uganda; olum.ronald@gmail.com; 2 Department of Medical Microbiology, School of Medicine, College of Medical Sciences, University of Benin, Benin City PMB 1154, Nigeria; iyabo.osaigbovo@uniben.edu; 3 Division of Pulmonology, Mulago National Referral Hospital, Kampala P.O Box 7272, Uganda; bbjoe18@gmail.com; 4 Makerere University Lung Institute, Kampala P.O. Box 7749, Uganda; 5 Excellence Center for Medical Mycology (ECMM), Department I of Internal Medicine, Faculty of Medicine and University Hospital Cologne, University of Cologne, Kerpener Str. 62, 50973 Cologne, Germany; jannik.stemler@uk-koeln.de; 6 Translational Research, Cologne Excellence Cluster on Cellular Stress Responses in Aging-Associated Diseases (CECAD), Faculty of Medicine and University Hospital Cologne, University of Cologne, Herderstr. 52, 50931 Cologne, Germany; 7 German Centre for Infection Research (DZIF), Partner Site Bonn-Cologne, Herderstr. 52, 50931 Cologne, Germany; 8 Translational Research Laboratory, Infectious Diseases Institute, College of Health Sciences, Makerere University, Kampala P.O. Box 22418, Uganda; kwizerarichard@ymail.com; 9 Department of Medical Microbiology and Immunology, Faculty of Medicine, Gulu University, Gulu P.O. Box 166, Uganda

**Keywords:** chronic pulmonary aspergillosis, *Aspergillus*, tuberculosis, Africa

## Abstract

Africa has a high burden of tuberculosis, which is the most important risk factor for chronic pulmonary aspergillosis (CPA). Our goal was to systematically evaluate the burden of CPA in Africa and map it by country. We conducted an extensive literature search for publications on CPA in Africa using the online databases. We reviewed a total of 41 studies published between 1976 and 2021, including a total of 1247 CPA cases from 14 African countries. Most of the cases came from Morocco (*n* = 764, 62.3%), followed by South Africa (*n* = 122, 9.9%) and Senegal (*n* = 99, 8.1%). Seventeen (41.5%) studies were retrospective, 12 (29.3%) were case reports, 5 case series (12.2%), 5 prospective cohorts, and 2 cross-sectional studies. The majority of the cases (67.1%, *n* = 645) were diagnosed in men, with a median age of 41 years (interquartile range: 36–45). Active/previously treated pulmonary tuberculosis (*n* = 764, 61.3%), human immunodeficiency virus infection (*n* = 29, 2.3%), diabetes mellitus (*n* = 19, 1.5%), and chronic obstructive pulmonary disease (*n* = 10, 0.8%) were the common co-morbidities. Haemoptysis was the most frequent presenting symptom, reported in up to 717 (57%) cases. Smoking (*n* = 69, 5.5%), recurrent lung infections (*n* = 41, 3%) and bronchorrhea (*n* = 33, 3%) were noted. This study confirms that CPA is common in Africa, with pulmonary tuberculosis being the most important risk factor.

## 1. Introduction

Chronic pulmonary aspergillosis (CPA) is an uncommon, slowly progressive pulmonary disease, most commonly caused by *Aspergillus fumigatus*, which presents with prominent respiratory and systemic symptoms associated with significant morbidity and mortality [1,2]. The diagnosis of CPA requires a combination of characteristics: chest imaging evidence of one or more cavities with or without a fungal ball present or nodules on thoracic imaging, direct microscopy/histopathology evidence of *Aspergillus* infection or an immunological response to *Aspergillus* spp., compatible symptoms present for at least three months and exclusion of alternative diagnoses [3,4].

CPA is increasingly being recognised as a global public health problem [5], with an estimated three million people affected, particularly those with underlying structural lung disease such as tuberculosis (TB), sarcoidosis, previous atypical mycobacterial infection, emphysema or those who have experienced a previous pneumothorax [6,7]. Of these, current or previous pulmonary tuberculosis (PTB) is the most common with prevalence ranging between 15% and 90% of patients with CPA [5,6]. CPA can mimic PTB and is often misdiagnosed, occurs during treatment of active PTB, or most often complicates PTB, especially in patients left with residual cavities [5,8].

Despite the high mortality associated with untreated CPA, that is, a 1-year mortality of ~20% and a 5-year mortality of ~75% [2,9], it is not currently considered by the World Health Organization (WHO) as a Neglected Tropical Disease (NTD) [10] since its burden is not well described. Africa has a high burden of TB and is therefore likely to have a high burden of CPA, but there are limited epidemiological studies to support this claim. Therefore, this study aimed to systematically review published cases of CPA and map its burden by country to inform policy, clinical care, and public health strategies.

## 2. Materials and Methods

### 2.1. Study Design

We conducted this systematic review in accordance with the Preferred Reporting Items for Systematic Review and Meta-analysis [11]. All databases were searched from inception to 31 July 2021.

### 2.2. Search Strategy

With the help of an experienced medical librarian, a comprehensive literature search was performed for publications on CPA cases or series in Africa using online databases Medline (via PubMed), Embase, African Journals Online, Google Scholar and gray literature papers. The search engine used the key words and the detailed medical subject heading (MeSH) terms to identify all published papers: “chronic pulmonary aspergillosis,” “CPA,” “pulmonary aspergilloma,” “post-tuberculosis lung disease,” “pulmonary mycetoma,” “Africa,” or the individual names of each of the country in Africa. The Boolean operators “AND” and “OR” were used to combine 2 or 3 terms.

### 2.3. Selection Criteria

We included all published studies, including case reports, case series, epidemiological and other observational study designs reporting on primary data from across Africa. We did not apply any language restriction. No date limitation or any other search criteria were applied to avoid missing papers published in Africa.

We excluded review articles and cases with grossly missing data, such as demographic characteristic and how the diagnosis of CPA was made.

### 2.4. Data Extraction

Data on study authors, study location (country and region in Africa), study period, age, sex, clinical presentation, underlying comorbidities, or risk factors were extracted. Data extraction was performed independently by two authors (RO and IIO) and any discrepancies solved by discussions.

### 2.5. Statistical Analysis

Data analysis was performed using Microsoft Excel 365 and STATA 16.0 (StataCorp LLC, College Station, TX, USA). Categorical characteristics of studies (e.g., country, study design, gender) were summarised as frequencies and percentages. Age from respective studies was summarised as median and interquartile range. Individual cases of CPA were summed up to give an overall number of patients diagnosed with CPA in Africa. CPA cases were stratified by country, region, and sex.

## 3. Results

### 3.1. Search Results

**Figure 1:**
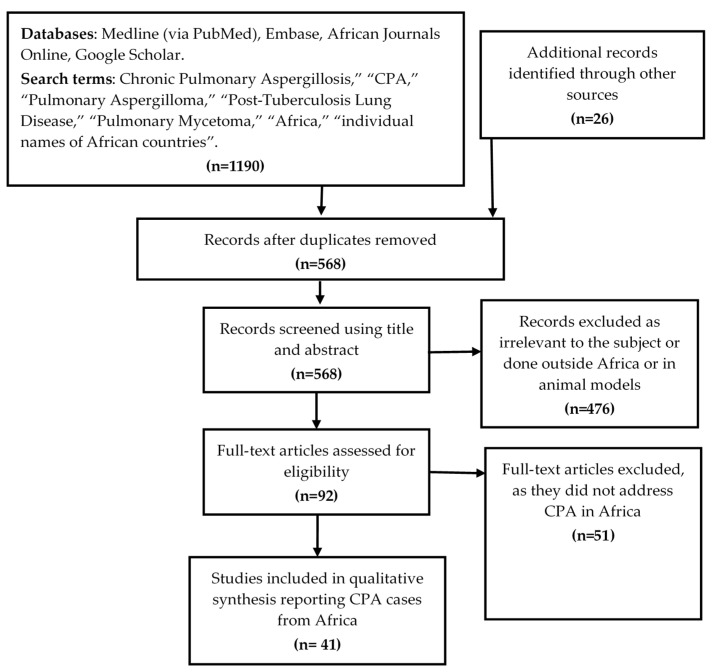
Citation selection process for the systematic review. Forty-one articles were found describing CPA cases in Africa.

Our initial database search retrieved 1190 publications and 26 identified from references of eligible studies. We then removed duplicates and 568 citations remained from which relevant studies were selected for review. Their potential relevance was examined using a title and abstract screening to remove studies that were clearly not related to scope of this review. A total of 476 citations were excluded as irrelevant to the subject. The full papers of the remaining 92 citations were assessed to select those that included primary data about CPA cases in any African country. These criteria excluded 51 studies and left 41 studies that were included in the final analysis (Figure 1).

### 3.2. Summary of Studies

We reviewed a total of 41 studies reporting CPA in 14 of the 54 African countries. (Table 1). Seventeen (41.5%) studies were retrospective chart reviews [12,13,14,15,16,17,18,19,20,21,22,23,24,25,26,27,28], 12 (29.3%) were case reports [29,30,31,32,33,34,35,36,37,38,39,40], 5 case series (12.2%) [8,41,42,43,44], 5 prospective cohorts [45,46,47,48,49], and 2 cross-sectional studies [50,51]. The majority of the studies were from East (*n* = 11) and West (*n* = 10) Africa. All studies were conducted between 1972 to 2019 and published between 1976 to 2021. Table 2 summarises the characteristics of the studies included in the review.

### 3.3. Cases of CPA in Africa

**Figure 2:**
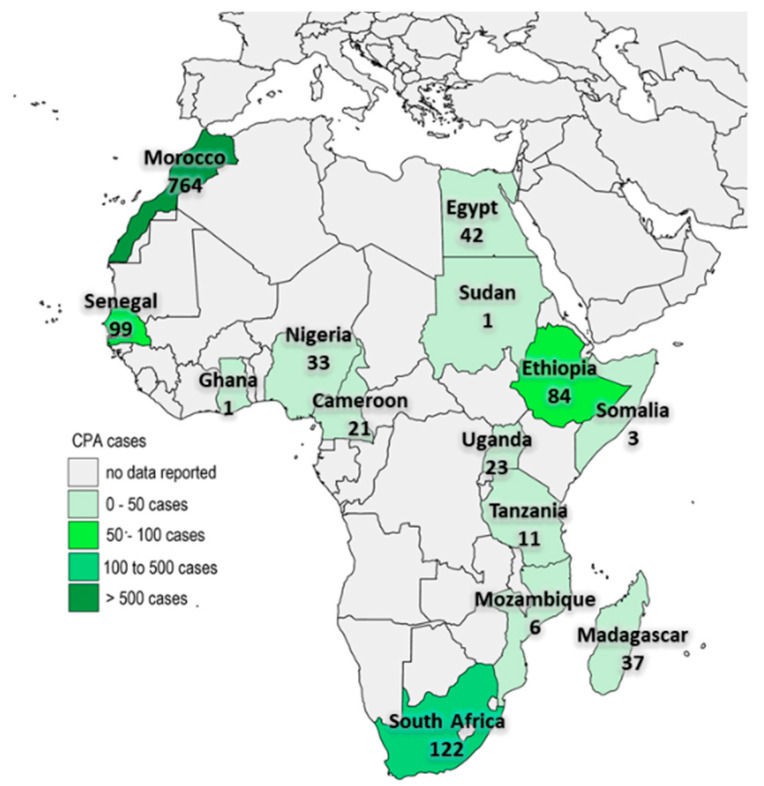
A map of Africa showing the distribution of CPA cases.

**Figure 3:**
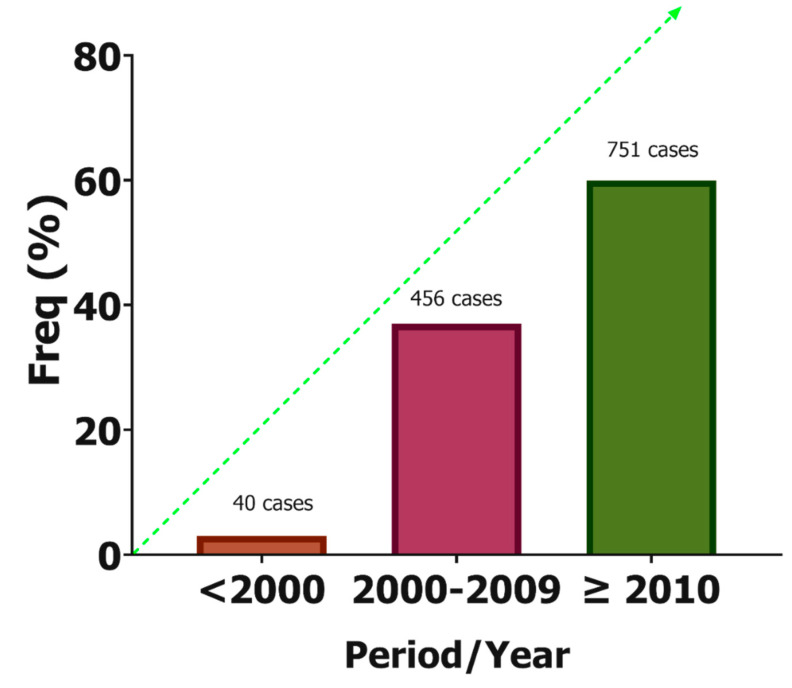
Trends in the report of CPA cases in Africa.

During the period of the review, 1247 cases of CPA were reported in Africa. Figure 2 shows the distribution of the reported cases in Africa. About one-third (67.1%, *n* = 645) were men and the median age was 41 years (interquartile range: 36–45 years). The majority of the reported cases were from Morocco (*n* = 764, 62.3%), followed by South Africa (*n* = 122, 9.9%) and Senegal (*n* = 99, 8.1%). When stratified by year of publication (≤1999, 2000–2009, and ≥2010), there has been an increase in the number of CPA reported in the literature over the past five decades (Figure 3).

**Figure 4:**
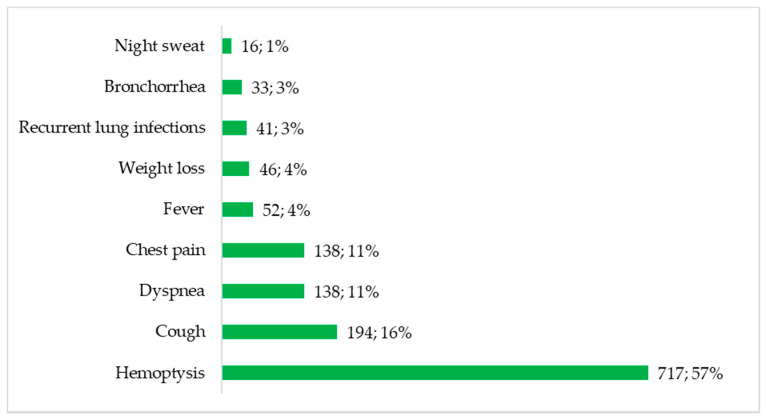
Presenting complaints of patients with CPA in Africa.

Figure 4 summarises the presenting complaints documented in the eligible studies. Haemoptysis was the most frequent presenting symptom/complaint, reported in up to 717 cases (57%). Cough (16%), difficulty in breathing (11%) and chest pain (11%) were also common symptoms. Recurrent lung infections (*n* = 41, 3%) and bronchorrhea (*n* = 33, 3%) were also noted.

### 3.4. Underlying Comorbidities

The most frequent underlying risk factors were active/previous tuberculosis (*n* = 764, 61.3%), smoking (*n* = 69, 5.5%) and HIV (*n* = 29, 2.3%). In fact, CPA was misdiagnosed as tuberculosis in two studies [8,32]. Other common comorbidities included bronchiectasis (*n* = 48, 3.8%), hydatid cysts (*n* = 19, 1.5%), diabetes mellitus (*n* = 19, 1.5%), lung abscess (*n* = 16, 1.3%), and COPD (*n* = 10, 0.8%). Pulmonary fibrosis (*n* = 1) [27] and lung malignancies (*n* = 2) [38,46] were infrequently reported.

## 4. Discussion

We present the first comprehensive attempt to enumerate all published CPA cases in Africa. Over 1200 CPA cases have been published over a 45-year period, with over 93% of the cases published after 2003—the year Denning and colleagues first described a set of diagnostic criteria for CPA [1]. In previous estimates of the burden of CPA, Agarwal and colleagues reported an annual incidence of CPA that varied between 27,000 and 0.17 million cases in the Indian sub-continent [52]. In addition, the global burden of CPA as a consequence of treated PTB has been estimated at 1.2 million cases [53] and approximately 72,000 cases as a sequela of sarcoidosis [54].

PTB was the most frequent underlying disease among the published cases. This is consistent with previously published cases from the rest of the world [6,9,55]. PTB and CPA share several important features—both being progressive parenchymal diseases with overlapping risk factors, symptoms and radiological findings that often lead to misdiagnosis of both disease [5,8,56]. In fact, in most low- and middle-income countries where the index of clinical suspicion and diagnostic capabilities are still low, CPA is managed as smear negative-TB.

Most CPA cases experienced haemoptysis and cough, which are also common symptoms in patients with PTB. This could explain why misdiagnosis for PTB was also common among these patients. The African patients were young and mostly men, findings which are consistent with reports from the United Kingdom [1], Europe [57], and Asia [55,58,59]. Therefore, across the world, CPA predominantly affects middle-aged men in the prime of their lives, and this is likely to have a negative impact on the global economy.

Recently, there has been tremendous advances in the diagnostics horizon for CPA, particularly the development of the *Aspergillus* IgG/IgM lateral flow assay for the serological diagnosis of CPA [60,61]. This has revolutionised the diagnosis of CPA in some low- and middle-income countries like Uganda [29], Mozambique [62] and Indonesia [63]. However, access to essential diagnostics for most fungal diseases and CPA in particular remains a challenge in many countries across Africa [64]. This could explain why only 14 of the 54 countries in Africa reported at least 1 case of CPA. The Global Action Fund for Fungal Infections (GAFFI) working together with its ambassadors globally, continues to advocate for increased access to and availability of essential diagnostics and drugs for major fungal diseases [65,66].

In this report, the prevalence of HIV infection (2.3%), and diabetes mellitus (1.5%) were both low. HIV does not appear to increase the predisposition to or prevalence of CPA among patients with active TB or those previously treated for TB in Africa [32,51]. Whether or not diabetes mellitus influences the incidence or prevalence of CPA in Africa remains unknown. In a recent study from Indonesia, 30% of patients with CPA had diabetes mellitus and patients with diabetes mellitus had a 6.7-fold the risk of developing CPA compared to non-diabetic populations [64]. Diabetes mellitus and HIV are also independent risk factors for sub-acute invasive aspergillosis and invasive aspergillosis which have poorer prognosis compared to CPA [67,68,69].

Untreated CPA can lead to progressive pulmonary fibrosis [70], progressive symptoms such as haemoptysis which may lead to high mortality [2], and a significant deterioration in the quality of life of those affected [71]. Early diagnosis of CPA, would allow an early start of antifungal treatment, usually given for at least 6 months, typically with oral itraconazole or voriconazole [3]. Follow up of TB patients for development of CPA is advocated and surveillance for CPA using point of care test should be built into the national TB control programmes for each African country to encourage early diagnosis and treatment [33].

Through advocacy by GAFFI, the point of care Aspergillus lateral flow assay was added to the WHO essential diagnostics list and should be able to help ease diagnosis of CPA in resource limited settings [66]. Oral itraconazole, the preferred antifungal for CPA, is available in at least 43% of African countries, but costly [72]. The cost varies widely from less than $1 in Uganda to $19 in Nigeria for a 400 mg/day dose. However, the WHO also recently added itraconazole on the “2017 Model List of Essential Medicines” for adults for management of selected fungal infections [73]. These efforts could reduce the cost of the diagnostics and treatment so thus encouraging screening and treatment programmes complemented with research studies in TB endemic areas.

## 5. Conclusions

In summary, we report substantial cases of CPA in Africa, especially in patients with currently active or previously treated PTB. Most of the patients are young and men. Therefore, CPA is an important, yet neglected disease that is significantly affecting economically dynamic people in Africa. Future research should focus on active screening for CPA in the most vulnerable populations.

## Figures and Tables

**Table 1:** Distribution of the studies included in the review.

Country	Region	Number of Publications	Percentage
Morocco [13,16,18,23,26,37,38,41]	North Africa	8	19.5
South Africa [15,22,27,35,47,48,49]	Southern Africa	7	17.1
Nigeria [14,28,32,34,50]	West Africa	5	12.2
Senegal [17,19,25,44]	West Africa	4	9.8
Uganda [8,29,30,51]	East Africa	4	9.8
Ethiopia [12,20,39]	East Africa	3	7.3
Cameroon [31,45]	Central Africa	2	4.9
Tanzania [24,36]	East Africa	2	4.9
Egypt [21]	North Africa	1	2.4
Ghana [33]	West Africa	1	2.4
Madagascar [46]	East Africa	1	2.4
Mozambique/Somalia [43]	East Africa	1	2.4
Somalia [42]	East Africa	1	2.4
Sudan [40]	North Africa	1	2.4

**Table 2:** Characteristics of studies reviewed.

	Study Type	Country	Region	Study Period	CPA Cases	Mean Age (Years)	Male: *n* (%)
Kwizera et al. (2021) [8]	Case Series	Uganda	East Africa	-	3	38.7	0 (0)
Alemu et al. (2020) [12]	Retrospective	Ethiopia	East Africa	2014–2019	72	35.2	46 (63.9)
Kwizera et al. (2020) [29]	Case Report	Uganda	East Africa	-	1	40.0	0 (0)
Harmouchi et al. (2019) [13]	Retrospective	Morocco	North Africa	2009–2018	79	40.5	57 (72.2)
Bongomin et al. (2019) [30]	Case Report	Uganda	East Africa	2018	1	45.0	1 (100)
Nonga et al. (2018) [31]	Case Report	Cameroon	Central Africa	-	1	47.0	1 (100)
Nonga et al. (2018) [45]	Prospective	Cameroon	Central Africa	2012–2015	20	30.0	17 (85)
Gbaja-Biamila et al. (2018) [32]	Case Report	Nigeria	West Africa	2016	1	35.0	1 (100)
Salami et al. (2018) [14]	Retrospective	Nigeria	West Africa	2014–2017	2	32.0	1 (50)
Masoud et al. (2017) [15]	Retrospective	South Africa	Southern Africa	2013–2015	59	46.6	36 (61)
Oladele et al. (2017) [50]	Cross sectional	Nigeria	West Africa	2014–2015	18	-	-
Page et al. (2019) [51]	Cross sectional	Uganda	East Africa	2012–2013	18	-	11 (80.0)
Issoufoua et al. (2016) [41]	Case Series	Morocco	North Africa	2009–2014	6	38.8	-
Ofori et al. (2016) [33]	Case Report	Ghana	West Africa	2013	1	38.0	1 (100)
Ekwueme et al. (2016) [34]	Case Report	Nigeria	West Africa	-	1	56.0	0 (0)
Hammoumi et al. (2015) [16]	Retrospective	Morocco	North Africa	2006–2014	274	37.8	93 (33.9)
Ba et al. (2015) [17]	Retrospective	Senegal	West Africa	2004–2008	35	43.4	28 (80)
Benjelloun et al. (2015) [18]	Retrospective	Morocco	West Africa	2003–2014	81	51.0	48 (59.3)
Koegelenberg et al. (2014) [35]	Case Report	South Africa	Southern Africa	-	1	30.0	1 (100)
Pohl et al. (2013) [36]	Case Report	Tanzania	East Africa	2011	1	68.0	1 (100)
Hammoumi et al. (2013) [37]	Case Report	Morocco	North Africa	-	3	47.7	3 (100)
Ade et al. (2011) [19]	Retrospective	Senegal	West Africa	2004–2008	35	43.4	28 (80)
Rakotoson et al. (2011) [46]	Prospective	Madagascar	East Africa	2006–2010	37	43.0	29 (78.4)
Smahi et al. (2011) [38]	Case Report	Morocco	North Africa	1991–2000	1	60.0	1 (100)
Gross et al. (2009) [47]	Prospective	South Africa	Southern Africa	-	10	41.4	-
Bekele et al. (2009) [20]	Retrospective	Ethiopia	East Africa	2005–2008	11	38.9	9 (81.8)
Brik et al. (2008) [21]	Retrospective	Egypt	North Africa	2001–2008	42	44.0	28 (66.7)
van den Heuvel et al. (2007) [22]	Retrospective	South Africa	Southern Africa	2001–2003	13	-	-
Hassan et al. (2004a) [42]	Case Series	Somalia	East Africa	2000–2003	1	-	-
Corr (2006) [48]	Prospective	South Africa	Southern Africa	2002–2003	12	36.0	9 (75)
Caidi et al. (2006) * [23]	Retrospective	Morocco	North Africa	1982–2004	320	32.0	161 (57.9)
Hassan et al. (2004b) [43]	Case Series	Mozambique /Somalia	East Africa	-	8	30.0	6 (75)
Falkson et al. (2002) [49]	Prospective	South Africa	Southern Africa	1989–1994	5	45.0	5 (100)
Mbembati et al. (2001) [24]	Retrospective	Tanzania	East Africa	1986–2000	10	-	8 (80)
Ba et al. (2000) [25]	Retrospective	Senegal	West Africa	1991–1998	24	-	-
Kabiri et al. (1999) * [26]	Retrospective	Morocco	North Africa	1982–1998	-	-	-
Aderaye et al. (1996) [39]	Case Report	Ethiopia	East Africa	-	1	25.0	0 (0)
Conlan et al. (1987) [27]	Retrospective	South Africa	Southern Africa	1982–1984	22	-	7 (31.8)
Adebayo et al. (1984) [28]	Retrospective	Nigeria	West Africa	1977–1983	11	42.2	7 (63.6)
Kane et al. (1976) [44]	Case Series	Senegal	West Africa	-	5	-	-
Mahgoub et al. (1972) [40]	Case Report	Sudan	North Africa	1968–1971	1	45.0	1 (100)

* Two studies, Caidi et al. (2004) [23] and Kabiri et al. (1999) [26], were from the same center and the latest was taken while computing the cases.

## Data Availability

Not applicable.
